# Proteomic profiles of underlying pathologies in genetic frontotemporal dementia

**DOI:** 10.1002/alz70856_105504

**Published:** 2026-01-10

**Authors:** Aitana Sogorb‐Esteve, Sophia Weiner, Joel Simrén, Imogen J Swift, Kaj Blennow, Henrik Zetterberg, Jonathan D. Rohrer, Johan Gobom

**Affiliations:** ^1^ Dementia Research Centre at University College London, London, United Kingdom; ^2^ UK Dementia Research Institute at University College London, London, United Kingdom; ^3^ Department of Psychiatry and Neurochemistry, Institute of Neuroscience and Physiology, The Sahlgrenska Academy, University of Gothenburg, Mölndal, Sweden; ^4^ Institute of Neuroscience and Physiology, Department of Psychiatry and Neurochemistry, The Sahlgrenska Academy at University of Gothenburg, Mölndal, Sweden; ^5^ Dementia Research Centre, Department of Neurodegenerative Disease, UCL Queen Square Institute of Neurology, University College London, London, United Kingdom, London, United Kingdom; ^6^ Institute of Neuroscience and Physiology, Sahlgrenska Academy at the University of Gothenburg, Gothenburg, Sweden; ^7^ University College London, London, United Kingdom, London, United Kingdom; ^8^ UK Dementia Research Institute at UCL, London, United Kingdom; ^9^ Dementia Research Centre, Department of Neurodegenerative Disease, UCL Queen Square Institute of Neurology, University College London, London, London, United Kingdom; ^10^ Clinical Neurochemistry Laboratory, Sahlgrenska University Hospital, Gothenburg, Gothenburg, Sweden

## Abstract

**Background:**

Although research has been prolific identifying proteomic changes in frontotemporal dementia (FTD), there are not strong evidence for the identification of biomarkers to help the understanding of the underlying pathologies. We explored proteomic profiles in genetic FTD associated to underlying pathologies.

**Method:**

This is a follow up study from a proteomic database in the GENetic FTD cohort (GENFI) in which a total of 238 cerebrospinal fluid (CSF) samples were measured by untargeted mass spectrometry. We performed ANCOVA analyses to identify the best predictors to differentiate among pathologies and then studied the pathways where these proteins are indicated.

**Result:**

Previous results from the study revealed individual proteomic changes in each genetic form of FTD. For the present work, we combined *C9orf72* and *GRN* mutation carriers to conform the TDP‐43 group and compared them against *MAPT* mutation carriers and controls. Among the proteins significantly altered in the disease groups compared to controls (155 in the TDP‐43 group and 52 in the tau group), 14 proteins were specific of *MAPT* mutation carriers but not on the others and 43 were only significantly changed in the TDP‐43 group. We identified DNASE2 and PLBD2 as potential predictors differentiating the underlying pathologies. Both proteins are significantly decreased in tau pathology, when compared to TDP‐43 pathology (DNASE2 *p*‐value<0.0001, PLBD2 *p*‐value=0.006). These two proteins are also the ones driving the significant changes in the cluster identified as “Lysosomal proteins” in our network analysis.

**Conclusion:**

Our results suggest a proteomic difference related to the underlying pathologies of FTD that, combined with the proteomic signatures linked to the genetic mutations, could provide key insights in the search for potential biomarkers for this disorder.

